# Predicting Functional Outcomes of Total Hip Arthroplasty Using Machine Learning: A Systematic Review

**DOI:** 10.3390/jcm13020603

**Published:** 2024-01-21

**Authors:** Nick D. Clement, Rosie Clement, Abigail Clement

**Affiliations:** 1 Edinburgh Orthopaedics, Royal Infirmary of Edinburgh, Little France, Edinburgh EH16 4SA, UK; 2 Southwest of London Orthopaedic Elective Centre, Epsom KT18 7EG, UK

**Keywords:** hip, arthroplasty, outcome, machine learning, PROMs, prediction, function

## Abstract

The aim of this review was to assess the reliability of machine learning (ML) techniques to predict the functional outcome of total hip arthroplasty. The literature search was performed up to October 2023, using MEDLINE/PubMed, Embase, Web of Science, and NIH Clinical Trials. Level I to IV evidence was included. Seven studies were identified that included 44,121 patients. The time to follow-up varied from 3 months to more than 2 years. Each study employed one to six ML techniques. The best-performing models were for health-related quality of life (HRQoL) outcomes, with an area under the curve (AUC) of more than 84%. In contrast, predicting the outcome of hip-specific measures was less reliable, with an AUC of between 71% to 87%. Random forest and neural networks were generally the best-performing models. Three studies compared the reliability of ML with traditional regression analysis: one found in favour of ML, one was not clear and stated regression closely followed the best-performing ML model, and one showed a similar AUC for HRQoL outcomes but did show a greater reliability for ML to predict a clinically significant change in the hip-specific function. ML offers acceptable-to-excellent discrimination of predicting functional outcomes and may have a marginal advantage over traditional regression analysis, especially in relation to hip-specific hip functional outcomes.

## 1. Introduction

The integration of artificial intelligence (AI) with the accumulation and storage of extensive data within electronic medical records has opened new possibilities for advancing orthopaedic research and its application in clinical settings [1]. The application of AI will likely play an essential role in the future to personalize patient treatment and aid recovery [2]. Machine learning (ML), a specific branch of AI, proves highly effective in handling the vast datasets at hand [3]. In the realm of orthopaedic surgery, particularly in total joint arthroplasty, ML is frequently employed for tabular data analysis (utilising spreadsheets), processing medical imaging, and employing natural language processing to extract concepts from textual information [4]. Various studies have explored ML models capable of discerning fractures in radiographs, identifying implant types in radiographic images, and determining osteoarthritis stages based on walking analysis [3,4,5,6]. Despite the increasing popularity of ML, it is important to acknowledge its limitations, such as its dependence on high-quality data, the potential for overfitting, a prolonged life cycle for development, and its capacity to address only specific, narrow tasks.

ML is a growing field in predicting the outcomes of patients, but it is not clear how reliable it is or whether it offers any advantages over traditional regression analysis [1]. Previous systematic reviews assessing the impact of ML on patient-reported outcomes following arthroplasty are limited and have included both total hip arthroplasty (THA) and knee arthroplasty [7,8]. A recently published review identified only three studies that assessed the reliability of ML to predict patient-reported outcomes following THA [7]. THA is a successful intervention for end-stage arthritis of the hip [9], being described as the most successful operation of the last century [10]. However, THA has limitations, and approximately 7% [11] to 12% [12] of patients are not satisfied with their hip. Numerous factors have been identified to be associated with dissatisfaction following THA using regression analysis to adjust for confounding factors [13].

ML models are capable of handling variable selection and interactions autonomously [3]. Traditional regression models are often designed to identify associations rather than focusing on predictive capabilities [3]. The distinctions between ML and traditional methods range from categorising anything outside traditional regression as ML to a continuum from traditional statistical models to ML, where a model is considered closer to ML when it requires less human input [3]. Whether ML models offer an advantage of traditional regression modelling is not clear [7,8].

The information from this review aims to inform clinicians of the current reliability of ML techniques and whether they should be employed in daily clinical practice to help inform patients of their potential functional outcomes after THA. Furthermore, this review aims to assess the reporting of the methodology used in the ML models and whether they were externally validated. This will enable the clinician to either be sceptical or be reassured by the predictability of the identified models.

## 2. Materials and Methods

This review was registered with PROSPERO (CRD42023446286) [14] and was carried out in line with the Preferred Reporting Items for Systematic Review and Meta-Analysis (PRISMA) statement [15]. The PRISMA checklist is included as Supplementary Material. The literature search was performed from January 1990 to October 2023, using the following databases: MEDLINE/PubMed, Embase, Web of Science, and NIH Clinical Trials. The search was limited to English-language papers. The search strategy used keywords “(artificial intelligence OR machine learning) AND (arthroplasty) AND (hip)” and was developed with the help of an experienced librarian. The Medical Subject Headings (MeSH) searches included “(artificial intelligence OR machine learning OR supervised machine learning OR neural networks) AND (hip arthroplasty OR hip replacement)”. Only studies with level I to IV evidence were included. Editorial, letters, conference papers, animal models, and abstracts were excluded from the study.

The control/comparator in ML studies is often termed the “Test” group, where the cohort is split into two groups, often an 80/20 split, and one group is used to “Train” the model (80%) and the other is used the “Test” the model (20%). These were included when reported by the identified studies. In addition, ML models are often compared to traditional regression modelling to assess the abilities of each method to predict outcomes, and this was reported also. This reliability of models to predict the outcome is often reported as the area under the receiver operating characteristic curve (AUC).

The aim of this systematic review was to assess the ability of ML to predict postoperative patient-reported outcome measures (PROMs) following primary THA performed for end-stage arthritis. Both hip-specific outcomes (Oxford hip score (OHS), hip disability and Osteoarthritis Outcome Score (HOOS)) and generic health-related quality of life assessments (short form (SF)-36, EuroQol (EQ) 5 dimension (D), and the visual analogue scale (VAS)) were included and assessed. The area under the receiver operating characteristic curve was used to assess the reliability of the ML models to predict patient-reported outcomes, and when compared to traditional regression analysis, this was reported. In addition, where external validation (a different cohort from that used to create and test the model) was undertaken, this was reported.

Two researchers (RC and AC) conducted the literature screening independently. Any disparity between the reviewers was decided by a third reviewer (NC). COVIDence software was used to facilitate the title/abstract screening, full-text review, and data extraction processes. The data extracted included the following: the authors, year of publication, size of cohort, level of evidence, variables included in the model, outcome measures employed, ML technique(s) used, handling of missing data, how the model was trained and tested, performance (AUC), whether it was compared to regression modelling or not, and whether it was externally validated. This was collated in an Excel spreadsheet from the studies identified.

Using the National Institutes of Health Quality Assessment Tool for Observational Cohort and Cross-Sectional Studies, all included publications were reviewed independently for a potential risk of bias. The assessment tool uses 14 questions to enable the allocation of a score to each article (poor, fair, or good). If there was disagreement regarding the scoring of a study, a consensus was met after a discussion amongst all authors.

Simple descriptive synthesis was undertaken. This focused on the reliability of the ML techniques to predict outcomes. This is reported as an AUC, where 0.5 equates to no discrimination, 0.5 to 0.7 has poor discrimination, 0.7 to 0.8 has acceptable discrimination, 0.8 to 0.9 has excellent discrimination, and more than 0.9 has outstanding discrimination [16]. Meta-analysis was planned to be undertaken to assess the overall predictability of machine learning models (combined AUC and 95% confidence intervals (CIs)) in the published protocol registered with PROSPERO, but this was not possible due the limited reported 95% CI of the included studies.

## 3. Results

**Figure 1:**
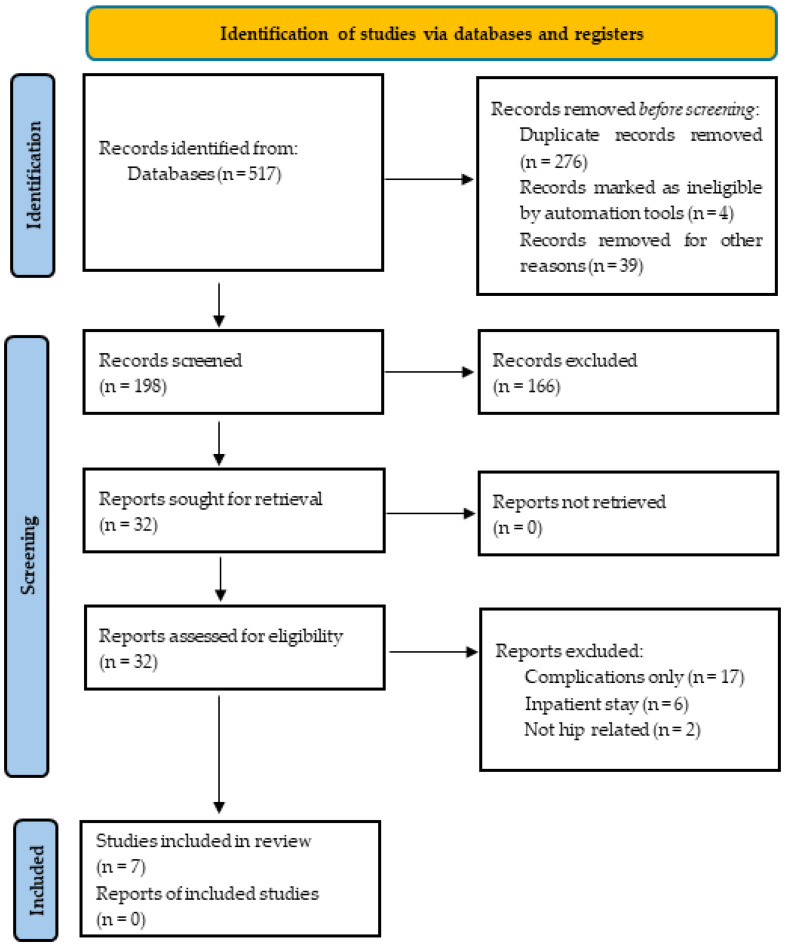
PRISMA flow diagram of the search strategy and included studies.

The systematic review identified seven studies that met the inclusion criteria, which included 44,121 patients (Figure 1) [17,18,19,20,21,22,23]. Sniderman et al. [21] employed slightly different methodology in their study compared to the other six studies and used ML to identify variables associated with postoperative hip-specific function but then assessed the reliability of these using logistic regression. Whereas the other six studies used ML models to identify variables associated with outcome and to predict patient-reported outcomes [17,18,19,20,22,23]. Six of the seven studies were published from 2019 onwards [17,18,19,21,22,23], with Schwartz et al. [20] publishing their study in 1997.

The sample sizes varied from 160 [21] patients to 31,905 [18] patients, with three studies having fewer than 1000 patients [19,20,21]. There was variation in the outcome measures employed to assess patient-reported outcomes, which included both joint-specific function and health-related quality of life assessments (Table 1). The search did not identify a study that had assessed patient satisfaction with their THA following surgery. The timepoint of the assessment varied from 3 months [21] to more than 2 years [19] following surgery. Three studies assessed improvements in the PROMs [20,21,22], and the remaining four assessed achievements of a clinically important improvement in the PROM [17,18,19,23]. However, the definition of clinically important varied between the studies (Table 1).

There were numerous ML techniques employed, and in addition, for comparison, three studies also used logistic regression analysis [18,20,23]. However, another also included regression analysis, but it was not for comparative purposes [21]. The number of ML techniques included in each study varied from one to six (Table 1). The four most commonly applied algorithms were neural networks (*n* = 5), random forest (*n* = 5), support vector machine (*n* = 3), and LASSO (*n* = 3). The majority of the models were assessed according to the AUC on the test dataset, with the exception of Huber et al. [18], who only reported the AUC for their training dataset. The test/train ratio also varied, with three studies using an 80:20 ratio for their data [17,22,23], one using a 2/3 and 1/3 ratio [21], another using a 70:30 ratio [19], and another using an approximately 50:50 ratio [18]. In the study by Schwartz et al. [20], the ratio of the test/train data was not clear.

The studies [17,19,20,21,22,23] reported acceptable (70–80% AUC) to outstanding (>90%) discrimination using their test data, with the exception of Huber et al. [18] who only reported this for the training dataset, which can lead to bias due to overfitting of the model [3,24]. The best-performing models were reported by Kunze et al. [19], who reported a >90% AUC for predicting an improvement in health-related quality of life using the minimal clinical important difference (MCID) in the EQ-VAS at a minimum of 2 years following THA. This is supported by Langenberger et al. [23], who found an 84% AUC in predicting a clinically significant improvement in the EQ-VAS at one year. In contrast, for those studies assessing hip-specific PROMs, the models were less reliable, with an AUC of between 71% [23] and 87% [22]. The study by Klemt et al. [22] reported a hip-specific outcome measure (HOOS), but it was not clear in the results as to which outcome measure their reported AUC related to as they also assessed three additional outcome measures. Random forest and neural networks were generally the best-performing ML models (Table 1). Three studies also included traditional regression analysis for comparison of the reliability of predicting outcomes, one found in favour of ML [20], and one stated that regression closely followed the best-performing ML model [18]. The third study demonstrated similar AUCs for EQ-5D and EQ-VAS for ML models and logistic regression [23] but did show a greater reliability for predicting a clinically significant change in the hip-specific function for ML models (neural network, ridge and elastic net) when compared to traditional logistic regression analysis. No study reported external validation of their model outwith their test/train data.

Some studies reported variables that influenced the predictive power of the models. Fontana et al. [17] demonstrated that the baseline PROM was either the first (SF36 mental and physical) or second (HOOS) most predictive feature included in their LASSO model. This was supported by the results from Klemt et al. [22] and Kunze et al. [19], who found the preoperative PROM to be the most important feature in their models. Langenberger et al. [23] also found the preoperative PROM scores to be the most important feature to predict the outcome PROM for achievement of MCID in the EQ-5D, EQ-VAS, and HOOS. The study by Sniderman et al. [21] aimed to use ML to identify patient-specific factors associated with hip-specific function following THA but, unlike the other studies, did not use ML to predict outcomes and used logistic regression to do so. Nonetheless, they found that frequent thoughts of work, frequent comparisons to healthier peers, increased body mass index (BMI), increased medical comorbidities, and the anterior surgical approach were associated with a worse 3-month HOOS score [21], whereas a better HOOS score was associated with employment at the time of surgery, thoughts related to family interaction, trying not to complain, and helping others [21].

There were several different strategies reported to deal with missing data (Table 1). Fontana et al. [17] handled numeric missing variables by imputation of the mean, while for categorial variables, an extra class was created for missing values. Huber et al. [18] removed patients with missing values and variables with variance close to or at zero. Kunze et al. [19] performed multiple imputation for variables with less than 30 percent missing values and excluded one variable with more than 30 percent missing data. Schwartz et al. [20] used the mean value of the missing variable. It is not clear in the study by Klemt et al. [22] how missing data were handled, and the study by Sniderman et al. [21] had less than 5% missing data, but again, it is not clear how these were handled. Langenberger et al. [23] used missForest imputation for data that were missing less than 30%.

Risk of bias was assessed using the National Institutes of Health Quality Assessment Tool for Observational Cohort and Cross-Sectional Studies, and no study was identified as poor-quality. Fontana et al. [17], Kunze et al. [19], and Langerberger et al. [23] were thought to be of good quality. Although they reported the largest series of patients, Huber et al. [18] removed those with missing data and only reported the reliability of the ML models for their training data. Both Schwartz et al. [20] and Sniderman et al. [21] used study cohorts with limited numbers of patients, 221 and 160, respectively, and the test/train ratio was not reported by Sniderman et al. [21]. Klemt et al. [22] did not describe how they handled their missing data.

## 4. Discussion

This review has shown that ML offers acceptable-to-excellent discrimination of predicting functional outcomes and may have a marginal advantage over traditional regression analysis, especially in relation to hip-specific hip functional outcomes. The best-performing models were for health-related quality of life (EQ-VAS and EQ-5D), with an AUC of more than 84%. In contrast, for those studies assessing hip-specific PROMs, the predictability of these models was less reliable, with an AUC of between 71% to 87%. Of the three studies that also included traditional regression analysis for comparison of the reliability of predicting outcomes, one found in favour of ML, one was not clear and stated that regression closely followed the best-performing ML model. The thrid showed no difference in reliability for EQ-5D and EQ-VAS but did show a greater reliability with ML in predicting a clinically significant change in the hip-specific function when compared to traditional logistic regression analysis. Of the studies reporting factors influencing their models, all found that preoperative PROMs were the most important variables associated with predicting outcome PROMs.

The limitations of this review should be acknowledged. The original aim in the registered protocol was to undertake a meta-analysis of the AUC and compare the reliability of ML with traditional regression analysis. However, this was not possible due to limited reporting of the 95% CI in addition to variation in the outcome measures used and definitions of clinically significant changes. Furthermore, only three studies reported the reliability of regression analysis in their cohort [18,20,23]. The authors suggest that future studies should report the 95% CI for the AUC to aid with this comparison. Secondly, although each ML model has specific techniques, the model parameters are at the discretion of the researcher, and, therefore, despite the same model being used, it may process those data in a different way. This may lead to some ML models performing better than others. In the studies reviewed, there was no control over this “hyperparameter tuning” [25], relying instead on the subjective judgment of individual researchers conducting the studies. Consequently, the authors recommend the inclusion of multiple specifications of the utilized models, with emphasis on indicating the best-tuned model. The models may also be influenced by missing data and how these are handled. The seven studies handled this differently, with some not stating how they had accounted for this and other studies employing multiple imputation techniques to address their missing data (Table 1). Finally, variables such as implant type, surgical approach, and mode of fixation, which may influence functional outcomes, were often not declared and, therefore, may be a limitation of the included models.

Four of the seven studies included defined the MCID for the PROMs assessed as their primary outcome. However, the MCID could be considered to be slightly different to the minimal important change (MIC), both of which are terms used in clinical research to describe the smallest change in a clinical measure that is considered meaningful or important to patients [26]. While the terms are closely related, they are sometimes used interchangeably, and their specific definitions can vary based on the context [27]. The MCID can be used to define smallest change in the measure between two patient groups, for example, group x versus y, whereas the MIC can be used to define a change in an individual’s or cohort’s score that is perceived to be a clinical benefit, for example, pre- to postoperatively [28]. The difference in these values does vary, with the MIC often being slightly greater than the MCID [26,27,28]. In future, studies may consider the MIC as an alternative to predicting the outcome THA as this may more likely represent a clinical benefit for an individual patient postoperatively relative to their preoperative baseline. Furthermore, there were also variations in the definition of the MCID between studies, with some using predefined values and others using half the SD. These differences may also influence the reliability of the models in predicting outcomes.

All studies that declared which variables influenced their models identified the patient’s preoperative functional status to be the greatest predictor of outcomes, which is common to traditional regression models [29,30]. Although it was not clear from the reviews, it would seem that the preoperative PROM was used as a total score. However, most of the PROMs assessed, with the exception of the EQ-VAS, can be broken down into the individual responses to each question. For example, the OHS has 12 questions that include both pain- and function-specific questions [31]. Due to the collinearity of these questions, inclusion of the individual responses in traditional regression models is problematic; however, in the knowledge than ML can adapt to this limitation, inclusion of the specific responses to each question may further improve the predictability of the models [32]. The reliability of the models across all of the studies was greater for the HRQoL PROMs compared to the hip-specific PROMs. It is not clear why this would be the case, but it may relate to the fact that the hip-specific outcome measures include both pain and functional measures, which may not be directly linked.

There have been several studies assessing the use of ML in predicting satisfaction after knee arthroplasty [7], but the authors did not identify any such studies assessing satisfaction after THA. Patient satisfaction following THA has been shown to be influenced by several factors, encompassing patient expectations, pain management, age, gender, comorbidities, and the duration of hospital stay [13]. It is estimated that between 7% [11] and 12% [12] of patients following THA are dissatisfied. Surgeons aiming to enhance satisfaction scores after THA may find an avenue for improvement by narrowing the gap between their expectations and that of their patients [12]. Using ML to help identify patients at risk of dissatisfaction may aid patient-specific decision making preoperatively with the knowledge of their predicted outcome and present them with realistic expectations.

Evidence-based medicine enables surgeons to trust that research findings will translate into benefits for their patients. However, a lack of understanding can alter this relationship [33]. Farrow et al. [5] proposed that when engaging with AI research, surgeons essentially assume the role of laypersons, likely due to the specialised nature of AI research methodology. Martin et al. [34] also highlighted a knowledge gap among surgeons regarding AI research that may lead to limitations in its impact on orthopaedics. Distinguishing between accepting and adopting research is crucial, with a noted delay of nearly two decades between research publication and widespread adoption [35]. The concept of “explainable AI” is underscored by the need for AI to be comprehensible, which was coined by the USA Defense Advanced Research Project Agency with the aim to “open the black box and let users see how conclusions were drawn” [36]. The term “black box” refers to a system where users see inputs and outputs but lack insight into its inner workings. This metaphor emphasizes the importance of transparency, aligning with trust, especially in healthcare. Samek et al. [37] cautioned against trusting predictions from a “black box” system without understanding its workings, deeming it irresponsible. It is also not clear how generalizable the results from the ML models are, with no study in the review validating their model using data from an external source. Therefore, to trust in the black box system without external validation may not be appropriate currently.

## 5. Conclusions

ML as part of AI would seem to be the future of orthopaedics to help inform patients of their potential outcome following THA. ML offers acceptable-to-excellent discrimination of predicting functional outcomes and may have a marginal advantage over traditional regression analysis, especially in relation to hip-specific functional outcomes.

## Figures and Tables

**Table 1:** Details and content of the seven included studies according to the defined areas of investigation.

Author, Year, and Evidence Level	Data Size	Data Source	Variables Included	Primary Outcome and Endpoint	Missing Data Handled	Test/Train Ratio	AI Models Used	Reliability Test Group (AUC)	Included Logistic Regression?	Better than Logistic Regression?	External Validation?
Fontana et al., 2019 [17] Level 3	7239	Single institution	Demographic *, medical, outcome scores	MCID at 2 years: SF-36 PCS (5.0), SF-36 MCS (5.0), HOOS Jr (17.7)	Imputation	80:20	LASSO, random forest, linear support vector machine	SF-36 PCS: 0.78 SF-36 MCS: 0.89 HOOS Jr: 0.78	No	Not Available	No
Huber et al., 2019 [18] Level 3	31,905	National Registry	Demographic *, outcome scores	MID at 6 months: EQ-VAS (11.0), OHS (8.0)	Removed prior to analysis	~50:50 (two separate years)	Extreme gradient boosting (EGB), multi-step elastic net, random forest, neural net, naïve Bayes, k-nearest neighbours	Only reported for training group EQ-VAS: 0.87 for EGB OHS: 0.78 for EGB	Yes	Not clearly stated but regression “followed closely” the best-performing model (EGB)	No
Kunze et al., 2020 [19] Level 3	616	Single institution	Demographic *, medical, preoperative health state	MCID at minimum 2 years: EQ-VAS (half standard deviation)	Multiple imputation (range of movement excluded missing >30%)	70:30	Random forest, stochastic gradient boosting, support vector machine, neural network, elastic net penalized logistic regression (ENPLR)	0.97 for random forest, 0.92 for neural network, 0.92 for stochastic gradient boosting, 0.90 for support vector machine, 0.87 for ENPLR	No	Not Available	No
Schwartz et al., 1997 [20] Level 3	221	Single institution	Demographic *, preoperative pain	Improvement in SF-36 pain at 1 year	Mean of missing variable	Not reported	Neural network	0.79	Yes	Yes (AUC: 0.79 vs. 0.74)	No
Sniderman et al. 2021 [21] Level 2	160	Single institution	Demographic *, medical, cognitive, surgical approach	3 months postoperative: HOOS	Less than 5%	67:33	LASSO	N/A	Yes	Not Available	No
Klemt et al., 2023 [22] Level 3	2137	Single institution	Demographic *, medical comorbidity, medications, surgical parameters	1 year postoperative: HOOS SF10A physical PROMIS physical PROMIS mental	Not stated	80:20	Random forest, support vector machine, neural network, elastic net–penalized logistic regression (ENPLR)	0.85 for random forest, 0.84 for support vector machine, 0.87 for neural network, 0.86 for ENPLR	No	Not Available	No
Langenberger et al., 2023 [23] Level 2	1843	Multicentre	Demographic *, activity level, outcome scores	MCID at 1 year: EQ-5D (0.2), EQ-VAS (5.86), HOOS (10.01)	<30% missing = imputed using missForest	80:20	Neural network, gradient boosting, LASSO, ridge, elastic net, random forest	Best-performing: EQ-5D: 0.81 EQ-VAS: 0.84 HOOS: 0.71	Yes	EQ-5D: 0.81 vs. 0.81 EQ-VAS: 0.84 vs. 0.84 HOOS: 0.71 vs. 0.67	No

* the demographics included in the models varied according to the study due to availability of these data, but generally included sex, age, BMI, and ASA grade.

## Data Availability

Data available upon reasonable request.

## Supplementary Materials

The following supporting information can be downloaded at: https://www.mdpi.com/article/10.3390/jcm13020603/s1, Prisma 2020 checklist.
